# Preparation and Characterization of *Tripterygium wilfordii* Multi-Glycoside Nanoparticle Using Supercritical Anti-Solvent Process

**DOI:** 10.3390/ijms15022695

**Published:** 2014-02-17

**Authors:** Fengli Chen, Tong Li, Shuangyang Li, Kexin Hou, Zaizhi Liu, Lili Li, Guoqiang Cui, Yuangang Zu, Lei Yang

**Affiliations:** State Engineering Laboratory for Bio-Resource Eco-Utilization, Northeast Forestry University, Harbin 150040, China; E-Mails: chenfengli1103@163.com (F.C.); styg12@126.com (T.L.); Rachel.0527@hotmail.com (S.L.); lynnhkx@hotmail.com (K.H.); zaizhiliu@hotmail.com (Z.L.); lilili001@126.com (L.L.); eric-cui1987@hotmail.com (G.C.); zygorl@163.com (Y.Z.)

**Keywords:** multi-glycoside, nanoparticle, supercritical antisolvent, physicochemical property, *Tripterygium wilfordii*

## Abstract

The aim of this study was to prepare nanosized *Tripterygium wilfordii* multi-glycoside (GTW) powders by the supercritical antisolvent precipitation process (SAS), and to evaluate the anti-inflammatory effects. Ethanol was used as solvent and carbon dioxide was used as an antisolvent. The effects of process parameters such as precipitation pressure (15–35 MPa), precipitation temperature (45–65 °C), drug solution flow rates (3–7 mL/min) and drug concentrations (10–30 mg/mL) were investigated. The nanospheres obtained with mean diameters ranged from 77.5 to 131.8 nm. The processed and unprocessed GTW were characterized by scanning electron microscopy, X-ray diffraction, Fourier-transform infrared spectroscopy and thermal gravimetric analysis. The present study was designed to investigate the beneficial effect of the GTW nanoparticles on adjuvant-induced arthritis in albino rats. The processed and unprocessed GTW were tested against Freund’s complete adjuvant-induced arthritis in rats. Blood samples were collected for the estimation of interleukins (IL-1α, IL-1β) and tumor necrosis factor-α (TNF-α). It was concluded that physicochemical properties and anti-inflammatory activity of GTW nanoparticles could be improved by physical modification, such as particle size reduction using supercritical antisolvent (SAS) process. Further, SAS process was a powerful methodology for improving the physicochemical properties and anti-inflammatory activity of GTW.

## 1. Introduction

*Tripterygium wilfordii* is a woody twining vine belonging to the family Celastraceae. It is native to Southern and Eastern China, Korea, and Japan [1]. It has been used in traditional Chinese medicine for over 2000 years [2], including treating fever, chills, sores, edema, carbuncle, joint pain, and inflammation [3,4]. Biochemical analysis has shown that *T. wilfordii* contains a mass of natural products with strong biological activities, one of the *T. wilfordii* root extracts was a stable glycoside, known as multi-glycoside of *T. wilfordii* (GTW) [1,5]. GTW has been approved by the China Food and Drug Administration for the treatment of rheumatoid arthritis and nephritis. The main bioactive components of GTW, such as triptolide, tripdiolide, triptonide, and triptohairic acid, were subjected to standardization. In recent years, it has also attracted much interest from scholars from various countries due to its anti-inflammatory [2,6,7], immunosuppressive [7,8], antitumor [9], antiviral effects [10], and its therapeutic impact on rheumatoid arthritis [11] and nephritis [12]. However, its long-term and large-dosing administration was restricted by the low solubility of its useful components.

Some factors such as membrane permeability and drug solubility limited the oral bioavailability, especially the solubility of active pharmaceutical ingredients in water, which was one of the most difficult and non-solved problems that often leads to insufficient bioavailability in pharmaceutical technology. The poor solubility of GTW leads to a low bioavailability, which causes a waste of resources and restricts their clinical use. Recently, in order to solve this problem, various GTW preparations have been researched, including microemulsion drug delivery system [13], GTW-polylactic acid nanoparticles delivery system [14], inclusion complexes [15], solid dispersion [16], and GTW liposome [17,18]. However, relatively large particle size, difficulties in complete recovery of organic solvents, or changes in chemical characteristics of drug limited the use of these methods and might cause uncertain side effects.

Supercritical fluid technology has been applied for chemical reaction [19], extraction [20], separation [21], coating [22], micronization [23], and so on. Particle formation by supercritical fluid technology has attracted attention due to its easy handling of difficult-to-comminute materials, using nontoxic medium, a mild operating temperature and providing ideal conditions for the processing of pharmaceutical compounds [24]. Moreover, there is no residual solvent in the final products, and no pollution to the environment [25]. Among various particle formation techniques using supercritical fluid which can be selected [26], the supercritical antisolvent (SAS) process was extensively used to prepare microparticles of organic and inorganic compounds [27–29]. Many researchers have employed the SAS process for micronization and recrystallization of various pharmaceutical substances [28,30,31].

Supercritical CO_2_ anti-solvent (SAS) process is an available technology to prepared drug nanoparticles, without the need for grinding procedure. This process was, briefly, the drug was firstly dissolved in the solvent and then the drug solution was quickly sprayed into supercritical fluids (the anti-solvent). Precipitation occurs immediately by a rapid recrystallization of the drug. Some operating parameters such as temperature, pressure, drug concentration, *etc*. have a great influence on particle size in SAS process [32,33]. Carbon dioxide is a widely used supercritical fluid because of its relatively low critical temperature, pressure and nontoxic property [34,35]. Moreover, CO_2_ is gaseous at ambient conditions, which simplifies the problem of solvent residues [36].

The purpose of this study was to prepare GTW nanoparticles by SAS process and evaluate their physicochemical properties and anti-inflammatory effects. To optimize the SAS micronization process, the effects of precipitation temperature, precipitation pressure, GTW solution concentration and solution flow rate on the mean particle size (MPS) of micronized GTW were studied by response surface methodology. Moreover, micronized GTW was characterized by scanning electron microscopy (SEM), Fourier-transform infrared spectroscopy (FTIR), X-ray diffraction (XR, and thermal gravimetric analysis (TG). The anti-inflammation effects of nanoscale GTW and non-nanoscale GTW were also evaluated by determining the changes of interleukin-1 (IL-1) and tumor necrosis factor-α (TNF-α) in complete Freund’s adjuvant–induced adjuvant arthritis in a Wistar rat model.

## 2. Results and Discussion

### 2.1. Effects of Precipitation Pressure, Precipitation Temperature, Drug Concentration, and Drug Solution Flow Rate on the MPS of GTW

**Figure 1.**
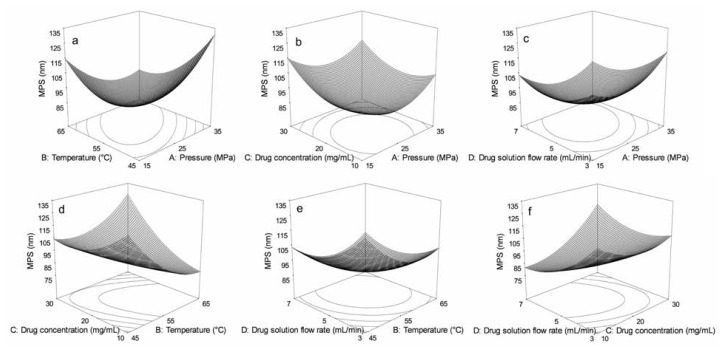
Optimization of preparation of GTW nanoparticles using BBD. (**a**) Interaction of precipitation pressure and temperature; (**b**) Interaction of precipitation pressure and drug concentration; (**c**) Interaction of precipitation pressure and drug solution flow rate; (**d**) Interaction of precipitation temperature and drug concentration; (**e**) Interaction of precipitation temperature and drug solution flow rate; (**f**) Interaction of drug concentration and drug solution flow rate.

Response surfaces were drawn to evaluate the effects of parameters and their interactions on MPS of GTW. The optimal values of the selected variables were obtained by solving the regression equation using the “Design Expert” software version 7.0. There were a total of 29 runs for optimizing the four individual parameters in the current Box–Behnken design (BBD), which was applied to the production of GTW nanoparticles. The values of responses (MPS of GTWs) at different experimental combination for coded variables were given in Table 1, from which we can see that the MPS ranged from 78.9 to 131.8 nm. The 3D response surfaces were graphical representations of the regression equation. Each contour curve represented an infinitive number of combinations of two test variables, keeping the other factors fixed at the zero level. Figure 1a shows that the 3D response surface of precipitation pressure (*X*_1_) and precipitation temperature (*X*_2_) with fixed drug concentration and drug solution flow rate (0 level). The MPS of GTW decreased as the precipitation pressure or precipitation temperature increased, and increased with further increases in the precipitation pressure or precipitation temperature. The 3D response surface for interaction of precipitation pressure (*X*_1_) and drug concentration (*X*_3_) with fixed precipitation temperature and drug solution flow rate (0 level) was shown in Figure 1b. We can see that the MPS of GTW decreased dramatically with the precipitation pressure increasing and remained constant with further increases in the precipitation pressure. The MPS of GTW decreased as the drug concentration increased and increased slowly as the drug concentration increased more. Figure 1c presents the 3D response surface of precipitation pressure (*X*_1_) and drug solution flow rate (*X*_4_) with fixed drug concentration and precipitation temperature (0 level), from which we can see that the MPS of GTW decreased and then increased slightly as the precipitation pressure or drug solution flow rate increased. Figure 1d shows that the 3D response surface of precipitation temperature (*X*_2_) and drug concentration (*X*_3_) with fixed precipitation pressure and drug solution flow rate (0 level). The MPS of GTW decreased significantly as the precipitation temperature increased. The MPS of GTW decreased and then remained constant with the drug solution flow rate increasing. The response surface for interaction of precipitation temperature (*X*_2_) and drug solution flow rate (*X*_4_) with fixed precipitation pressure and drug concentration (0 level) was shown in Figure 1e. We can see that the MPS of GTW decreased and then remained constant as the precipitation temperature and drug solution flow rate increased. Fig 1f presents the 3D response surface of drug concentration (*X*_3_) and drug solution flow rate (*X*_4_) with fixed precipitation pressure and precipitation temperature (0 level), from which we can see that the MPS of GTW decreased significantly as the drug solution flow rate increased. The MPS of GTW decreased and then remained constant as the drug concentration increased.

The optimum extraction conditions (*X*_1_ = 29.4 MPa; *X*_2_ = 63.5 °C; *X*_3_ = 10.2 mg/mL, and *X*_4_ = 5.4 mL/min) for the MPS of GTW were estimated using the model equation and by solving the regression equation. The theoretical MPS of GTW that was predicted under the above conditions was 74.7 nm.

### 2.2. Model Building and Statistical Analysis

The data obtained from all the experiments are summarized in Table 1. There were a total of 29 runs for optimizing the four individual parameters that determined MPS of GTW. By applying multiple regression analysis on the experimental data, the response variable and the test variables were related by the second-order polynomial Equation (1):

(1) $$\begin{array}{l} Y=741.39+2.92X_{1}-14.88X_{2}-15.15X_{3}-43.94X_{4}-0.13X_{1}X_{2}-0.02X_{1}X_{3}-0.21X_{1}X_{4}\\ +0.17X_{2}X_{3}+0.21X_{2}X_{4}+0.56X_{3}X_{4}+0.11{X_{1}}^{2}+0.11{X_{2}}^{2}+0.10{X_{3}}^{2}+2.29{X_{4}}^{2} \end{array}$$

The significance of each coefficient was checked using the *F*-test and the *p* value. The *p* value was used to check the significance of each coefficient and it also indicated the interaction strength between each independent variable. The analysis of variances of the quadratic regression model demonstrated that the model was significant, which was evident from the *F*-test low probability value (*p* = 0.0005). The 6.65 model *F*-value implied that the model was significant, since there was only a 0.05% chance that a value of this size could occur due to noise. The “lack of fit *F*-value” of 3.24 was not significant relative to the pure error. There was a 13.41% chance that a “lack of fit *F*-value” of this size could occur because of noise. Thus, the non-significant lack of fit showed the model was suitable for data analysis. The regression coefficients and the corresponding *p* values are also shown in Table 1. Values of “Prob > *F*” less than 0.0500 indicated that the *X*_2_, *X*_3_, *X*_4_, *X*_1_*X*_2_, *X*_2_*X*_3_, *X*_3_*X*_4_, *X*_1_^2^, *X*_2_^2^, *X*_3_^2^, and *X*_4_^2^ model terms were significant. Values greater than 0.1000 indicated model terms that were not significant, such as *X*_1_, *X*_1_*X*_3_, *X*_1_*X*_4_, and *X*_2_*X*_4_. The coefficient of determination (*R*^2^ = 0.8692), the adjusted coefficient of determination (*R_Adj_*^2^ = 0.7385) and the coefficient of variation (7.62%) are shown in Table 1. “Adeq precision” measured the signal-to-noise ratio. A ratio greater than 4 was desirable, thus, a ratio of 9.1724 indicated an adequate signal. This model can be used to navigate the design space.

### 2.3. Verification Tests

The verification tests were done three times under the conditions of point prediction by RSM (29.4 MPa precipitation pressure, 63.5 °C precipitation temperature, 10.2 mg/mL drug concentration, 5.4 mL/min drug solution flow rate). The actual MPS of GTW was 77.5 nm with an error of 2.8 nm, which conformed to theoretical MPS of GTW (74.7 nm).

### 2.4. Morphology of Micronized GTW

**Figure 2.**
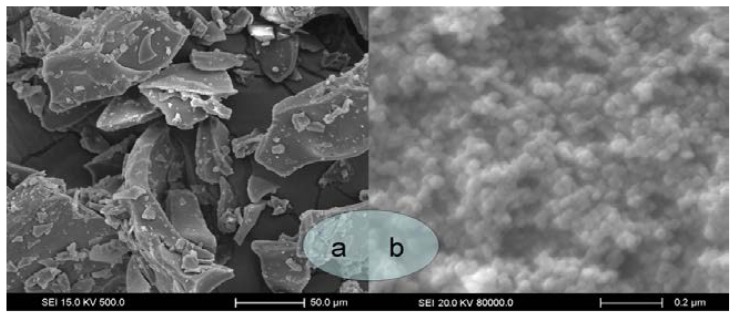
SEM images of unprocessed GTW (**a**), processed GTW was obtained under the optimum extraction condition: 29.4 MPa precipitation pressure, 63.5 °C precipitation temperature, 10.2 mg/mL drug concentration, 5.4 mL/min drug solution flow rate (**b**).

Figure 2a,b shows the SEM morphologies of GTW raw materials and GTW nanoparticles obtained by supercritical antisolvent precipitation (temperature was 63.5 °C, pressure was 29.4 MPa, the drug concentration was 10.2 mg/mL, the solution flow rate was 5.4 mL/min and the CO_2_ flow rate was 8.5 kg/h), respectively. MPS of nanoparticles has been previously determined using particle size analyzer laser (MPS was 77.5 nm). We can easily observe that most of the nanoparticles appeared spherical or subglobose, nonetheless, GTW raw materials exhibited an irregular shape, a micron-grade particle size and a wide size distribution. Consequently, SAS recrystallization is capable to produce regular and nanosized GTW particles, which might be able to enhance the oral absorption of drug powder.

### 2.5. Analysis of GTW and Triptolide

**Figure 3.**
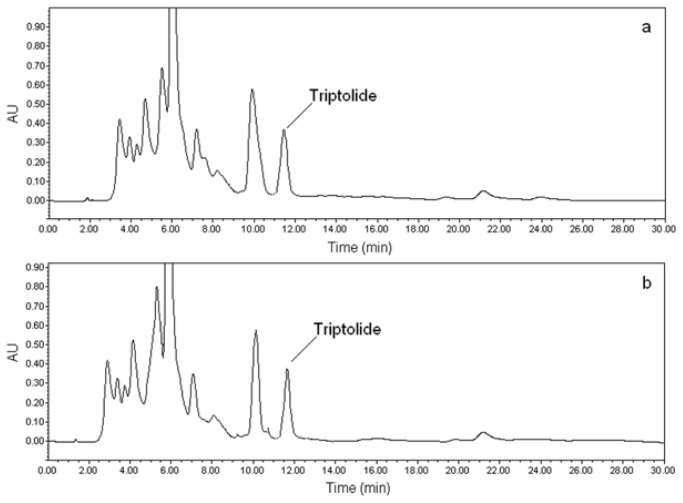
HPLC chromatograms of GTW. (**a**) unprocessed and (**b**) processed.

The contents of GTW and triptolide in unprocessed and processed samples were analyzed and determined using the method mentioned in Sections 3.11. and 3.12. The results are summarized in Table 2, from which it can be seen that the contents of GTW and triptolide in unprocessed and processed samples were not significantly different (*p* < 0.05). The HPLC chromatograms of unprocessed and processed samples are shown in Figure 3.

### 2.6. Characterization of GTW Nanoparticles

**Figure 4.**
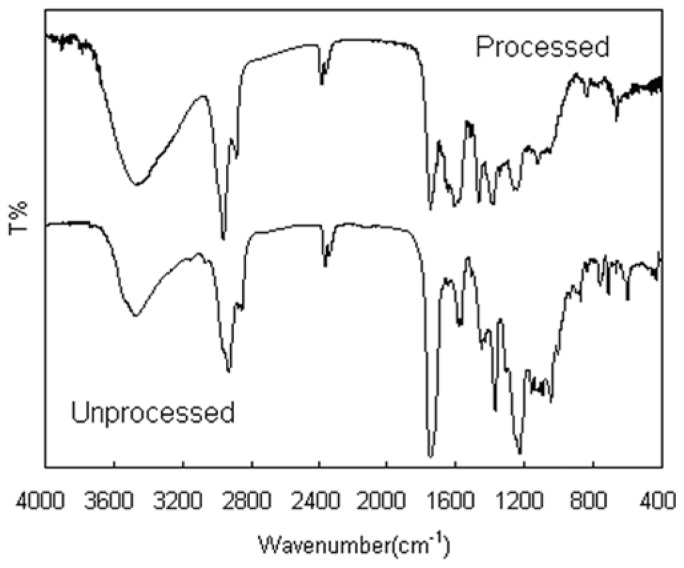
Comparative investigations on FTIR spectra of unprocessed and processed GTW.

We performed some analysis on unprocessed and processed GTW to obtain information on the changes of physical and chemical structures after SAS processing. The FTIR spectra of GTW raw materials and GTW nanoparticles in the range of 400–4000 cm^−1^ were compared in Figure 4. The assignments of function groups or chemical bonds were as follows: 3465 cm^−1^ (free O–H stretching vibrations), 2935 and 2840 cm^−1^ (C–H stretching vibrations), 1735 cm^−1^ (stretching vibration of carbonyl group), 1600 cm^−1^ (C–C stretching vibration of benzene ring), 1447, 1390, and 1218 cm^−1^, the positions and intensities of these peaks did not obviously change after SAS processing. It can be seen that FTIR spectra of unprocessed and processed GTW did not show any significant differences.

**Figure 5.**
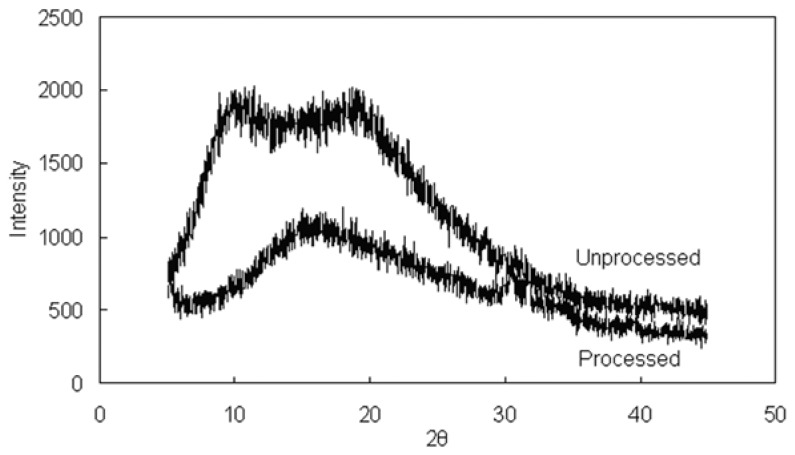
Comparative investigations on XRD patterns of unprocessed and processed GTW.

X-ray diffraction analysis of GTW raw materials and GTW nanoparticles was performed to investigate further the crystallinity of particles. Based on the results of GTW raw materials and GTW nanoparticles which are shown in Figure 5, no obvious diffraction peak with very high intensity were observed on the diffraction pattern of GA raw materials, but a lot diffraction peaks with weak intensity can be observed at the diffraction angles of 2θ = 5°–45°. This result revealed the unprocessed GTW has a certain degree of crystallinity, but the degree of crystallinity is weak. A lot weaker diffraction peaks with smaller intensity were seen in the XRD of processed GTW. This phenomenon indicated that the crystallinity of GTW decreased greatly and amorphous state of GTW was formed almost entirely by SAS. As far as drug particles are concerned, the amorphous form plays an important role in solubility, which usually results in a higher solubility.

**Figure 6.**
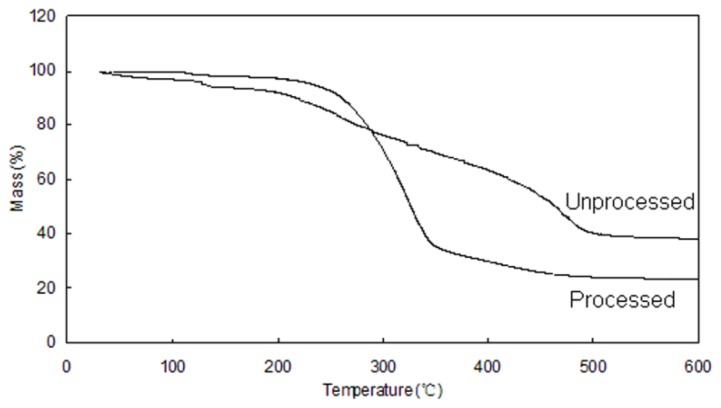
Comparative investigations on TG of unprocessed and processed GTW.

The TG curves of unprocessed and processed GTW are shown in Figure 6. There is a loss of water in the unprocessed drug below 100 °C and no loss of water can be observed in processed GTW. However, the processed GTW showed a higher weight loss compared with the unprocessed GTW between 250 and 350 °C. This may be due to the fact that the small particles possess a higher specific surface energy, which leads to an easier vaporization and decomposition.

Proinflammatory cytokines such as IL-1α, IL-1β, and TNF-α released from inflammatory foci initiate a local inflammatory response. Measuring the levels of these mediators of inflammation in the synovial fluid can provide information about the underlying pathophysiology of joint disease. In the present study, as shown in Table 3, synovial IL-1α, IL-1β, and TNF-α levels were significantly increased in FCA-induced experimental arthritis. Treatment with both doses of GTW (processed and unprocessed) showed a significant decrease in synovial IL-1α, IL-1β, and TNF-α levels. GTW nanoparticles had a more prominent effect on decreasing the synovial IL-1α, IL-1β, and TNF-α levels than GTW raw materials (6.2 ± 0.62, 9.9 ± 0.38 and 6.3 ± 0.54). Therefore, the SAS process was a powerful method for improving anti-inflammatory activity of GTW.

## 3. Experimental Section

### 3.1. Drugs and Chemicals

High purity CO_2_ (99.99% pure) was purchased from Liming Gas Company of Harbin (Harbin, Heilongjiang, China). Absolute ethanol (analytical grade) was purchased from Sinopharm Chemical Reagent Beijing Co., Ltd. (Beijing, China). Deionized water was prepared by a Milli-Q water purification system (Millipore, Bedford, MA, USA) and was used in all experiments. GTW was kindly provided by Jiangsu Meitong Pharmaceutical Co., Ltd. (Taizhou, China). Reference triptolide was purchased form National Institute for the Control of Pharmaceutical and Biological Products (Beijing, China). Freund’s Complete Adjuvant (FCA) was purchased from Sigma-Aldrich (St. Louis, MO, USA). All other solvents and chemicals used in this study were of analytical grade from Beijing Chemical Reagents Co. (Beijing, China).

### 3.2. SAS Apparatus

**Figure 7.**
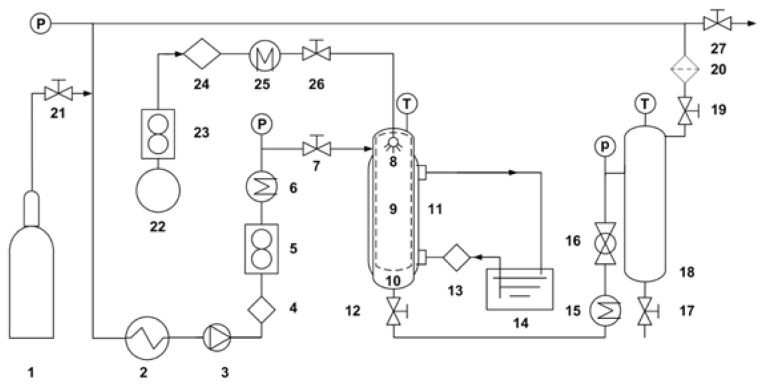
Schematic diagram of the experimental apparatus for the SAS process. 1, CO_2_ cylinder; 2, CO_2_ cooler; 3, CO_2_ pump; 4 and 24, liquid pump; 5 and 23, flowmeter; 6, 15 and 25, heat exchangers; 7, 12, 17, 19, 21, 26 and 27, valves; 8, nozzle; 9, stainless steel core vessel of 150 μm; 10, precipitation chamber; 11, jacket bath; 13, hot water circulating pump; 14, thermal bath; 16, back pressure valve; 18, gas-liquid separation chamber; 20, filter; 22, liquid solution supply.

The schematic diagram of the SAS process apparatus which is shown in Figure 7 mainly consisted of a CO_2_ cylinder (1), a stainless steel precipitation chamber (10) and a gas–liquid separation chamber (18). The CO_2_ was cooled with a cooler (2) before being compressed by a pump (3) and the pressure which could be read on pressure meter was controlled by a back pressure valve (16). The CO_2_ was preheated by a heat exchanger (6) and entered into the 200 nm of stainless steel core vessel (9) located in precipitation chamber (10). The temperature was measured by temperature meter and kept constant by a hot water circulating pump (13). When the CO_2_ supercritical conditions were achieved, the solution pump was started. The liquid solution was pumped by a liquid pump (24), heated by a heat exchanger (25) and fed to the precipitation chamber through a 150 μm diameter stainless steel nozzle (8). A stainless steel core vessel (9) located into the precipitation chamber was used to collect the produced powder and to let the SC-CO_2_/ethanol mixture pass through. The flow rate of the mixture that leaves the precipitation chamber was controlled by a valve (12) located between the precipitation chamber (10) and the gas-liquid separation chamber (18). The mixture suffered a decompression (pressure < 5 MPa) to separate the CO_2_ from the organic solvent in the gas–liquid separation chamber (18).

### 3.3. Animals

Female Wistar rats (weighing 100–200 g) were obtained from Shanghai Slac Laboratory Animal Co. Ltd., (Shanghai, China). They were kept at ambient temperature and had free access to water and diet. All animals received humane care and all experimental procedures abided by the ethics and regulations of animal experiments. Four groups of animals were formed, each group consisting of six rats.

### 3.4. Preparation of GTW Nanoparticles by SAS

There are many factors which affect the MPS of GTW, including precipitation temperature, precipitation pressure, GTW solution concentration, GTW solution flow rate, type of solvent, composition of solvent, pore diameter of nozzle and CO_2_ flow rate. This research investigated some important of these factors and the same solvent ethanol, 150 μm pore diameter of nozzle and 8.5 kg/h CO_2_ steady flow were constant and used for all SAS. During the SAS process, four main factors: pressure, temperature, drug concentration, and drug solution flow rate were studied by response surface methodology.

The SAS experiment begins by pumping supercritical CO_2_ into the precipitation chamber until the desired pressure and temperature were reached. The steady flow of CO_2_ was established at 8.5 kg/h. Then, pure solvent was sent through the liquid pump to the precipitation chamber to obtain steady state composition conditions during the solute precipitation. At the point, the flow of the pure ethanol was stopped and the GTW ethanol solution was pumped into the particle precipitation chamber by high pressure liquid pump through a 150 μm nozzle. The liquid pump was stopped when fixed quantity of GTW ethanol solution was injected. However, the supercritical CO_2_ continued to flow to wash the stainless steel core vessel with the aim of removing residual liquid solubilized into the supercritical antisolvent for 30 min at least. Finally, the supercritical CO_2_ flow was stopped and the precipitation chamber was then depressurized gradually to atmospheric pressure. The collected drugs were taken out of the stainless steel core vessel for further characterization analysis.

### 3.5. Optimization of SAS Process

BBD was employed to statistically optimize the formulation parameters and evaluate main effects, interaction effects and quadratic effects of the formulation ingredients on the MPS of processed GTW. Many factors affect the MPS of GTW, including precipitation temperature, precipitation pressure, GTW solution concentration, GTW solution flow rate, type of solvent, composition of solvent, pore diameter of nozzle, and CO_2_ flow rate. According to the principle of BBD, precipitation pressure (*X*_1_), precipitation temperature (*X*_2_), concentration of GTW solution (*X*_3_) and flow rate of GTW solution (*X*_4_) were chosen as the key variables based on the results of preliminary experiments, which were identified to have strong effects on the MPS, were taken as the variables tested in a 29-run experiment, and designated as *X*_1_, *X*_2_, *X*_3_, and *X*_4_, respectively, as shown in Table 1. The fluctuation ranges of the variables *X*_1_, *X*_2_, *X*_3_, and *X*_4_ were about 1 MPa, 2 °C, 0.1 mg/mL, and 0.2 mL/min, respectively. The fluctuation range of constant parameter (CO_2_ flow rate) was about 0.5 kg/h. Five replicates at the center of the design were used for the estimation of a pure-error sum of squares. Experiments were randomized to maximize the effects of unexplained variability due to extraneous factors, in the observed responses. A quadratic equation was used for this model as follows:

(2) $$Y=\beta_{0}+\sum_{i=1}^{4}\beta_{i}x_{i}+\sum_{i=1}^{4}\beta_{ii}x_{i}^{2}+\sum_{i=1}^{3}\sum_{j=i+1}^{4}\beta_{ij}x_{i}x_{j}$$

where *Y* is the estimated response, β_0_, β*_i_*, β*_ii_*, and β*_ij_* are the regression coefficients for the intercept, linearity, square and interaction, respectively, and *X*_1_, *X*_2_, X_3_, and *X*_4_ are the independent variables.

Analysis of the experimental design and data was carried out using Design-Expert (Version 7.0, Stat-Ease Inc., Minneapolis, MN, USA) software. ANOVA was performed, and the fitness of the polynomial model equation was expressed by the coefficient of determination *R*^2^. Its statistical significances were checked by *F*-test at a probability (*p*) of 0.001, 0.01 or 0.05. The significances of the regression coefficients were also tested by *F*-test.

### 3.6. Particle Size Analysis

The MPS of prepared nanoparticles was measured by DLS (ZetaPALS/90 plus; Brookhaven Instruments Corporation, Holtsville, NY, USA) particle size analyzer. Processed GTW was suspended in deionized water (no surfactant was used in water) and sonicated for 3 min at 100 W (KQ-100VDE, Kunshan Ultrasonic Instruments Co., Kunshan, China) to avoid the aggregation of particles. The whole process was operated on a clean workbench to prevent dust or other particulate transmission. The suspensions were analyzed in the DLS particle size analyzer. Every measurement was repeated at least three times at 25 °C with a temperature-controlled system. The MPS and standard deviations (SD) obtained were used to fit the particle size distribution to a lognormal distribution.

### 3.7. Scanning Electron Microscopy (SEM)

The surface morphological examinations of the unprocessed and processed GTW were conducted using a scanning electron microscope (FEI Quanta 200, Hillsboro, OR, USA). Particles of representative samples were fixed to an SEM stub with a carbon conductive and sputter-coated with gold using sputter-coater (KYKY SBC-12, Beijing, China) at room temperature before examination.

### 3.8. Fourier Transforms Infrared Spectroscopy (FTIR)

The FTIR spectrum was obtained by MAGNA-IR560 E.S.P spectrophotometer (Nicolet, Madison, WI, USA). The unprocessed and processed GTW were diluted with KBr mixing powder at 1% and pressed to obtain self-supporting disks, separately. Tablets for FTIR measurements were prepared by pressing the mixture powder at a load of 5 tons for 2 min and recorded in the wave number range of 400–4000 cm^−1^ at a resolution of 4 cm^−1^.

### 3.9. X-ray Powder Diffraction (XRD)

X-ray diffraction patterns were obtained in transmission using an X-ray diffractometer with a rotating anode (Philips, Xpert-Pro, Almelo, The Netherlands) with Ni-filtered Cu Kα1 radiation generated at a voltage of 40 kV and 30 mA. Powders of the unprocessed and processed GTW were filled to the same depth inside the sample holder by leveling with a spatula. The range of 2θ diffraction angle examined was 5°–45° with steps of 0.02° and a measuring time of 0.3 s per step.

### 3.10. Thermal Gravimetric Analysis (TG)

The thermal gravimetric analysis of samples was carried out with a Perkin-Elmer Pyris 1 TG instrument (Perkin-Elmer Co., Norwalk, CT, USA). The experiments were performed with a heating rate of 10 °C/min using nitrogen flow (150 mL/min) and unprocessed and processed GTW powers were weighed (approximately 5 mg) in open aluminum pans and the percentage weight loss of the samples were monitored from 35 to 600 °C.

### 3.11. Determination of GTW Using Kedde’s Reagent

The GTWs were determined using 3,5-dinitrobenzoic acid–NaOH method [37,38] and using triptolide as the standard. Briefly, a 0.047 mol/L solution of 3,5-dinitrobenzoic acid in ethanol was mixed in the ratio of 1:1 with a solution of 0.25 mol/L NaOH in distilled water. This mixture was mixed with 1 mL of unprocessed and processed GTW in ethanol. After vortexing for 5 min, the absorbance of the solution was measured using a UV spectrophotometer (UV-2550, Shimadzu, Kyoto, Japan) at 540 nm.

### 3.12. Determination of Triptolide by HPLC

The HPLC system consisted of a Waters 717 automatic sample handling system series HPLC system equipped with a 1525 Bin pump and a 2487 UV-detector (Waters, Milford, MA, USA). Triptolide in unprocessed and processed GTW was determined by HPLC [39]. Chromatographic analysis was carried out by Hypersil BDS C18 reversed phase column (5 μm, 4.6 mm × 250 mm) and methanol–water (40:60, *v*/*v*) was used as the mobile phase. The mobile phase was filtered through a 0.45 μm membrane filter (Guangfu Chemical Reagents Co., Tianjin, China) and then deaerated ultrasonically prior to use. Flow rate and injection volume were 1.0 mL/min and 10 μL, respectively. Analytical wavelength was set at 218 nm and all chromatographic operations were carried out at ambient temperature. The chromatographic peak of the analyte was confirmed by comparing its retention time with the reference standard.

### 3.13. Freund’s Complete Adjuvant-Induced Arthritis

Four groups, except the normal group, were made arthritic by injecting 0.1 mL Freund’s complete adjuvant into the subplantar region of the left hind paw on day “0”. Unprocessed or processed (10 mg/kg) was administered orally once daily, from the day of adjuvant injection (0 day), 30 min before adjuvant injection, and continued until the 21st day.

On day 22, blood was withdrawn from each animal through retro-orbital plexus puncture by anaesthetizing the animals with anaesthetic ether. The blood was collected into vials containing EDTA, and serum was obtained from 2 mL blood by 2000 rpm of centrifugation for 15 min for the tests of IL-1α, IL-1β, and TNF-α. Serum IL-1α, 1L-1β, and TNF-α concentrations were assayed using mouse cytokine and hormone ELISA test kits [40]. The concentrations of IL-1α, 1L-1β were determined using commercial ELISA kits (R&D Systems China Co. Ltd., Shanghai, China) according to the manufacturer’s instruction. The detection threshold was 3 pg/mL. The levels of TNF-α were measured using commercially available ELISA kits that specifically recognize the rat cytokines (R&D Systems China Co. Ltd., Shanghai, China) according to the manufacturer’s instruction. The minimum detection limit of the assay was 2 pg/mL.

### 3.14. Statistical Analysis

The data was subjected to analysis of variance and the significance of the difference between means was determined by Duncan’s multiple range test (*p* < 0.05) using SAS (Version 8.1, 2000; SAS Inst., Cary, NC, USA). Values expressed are means ± standard deviation.

## 4. Conclusions

In this work, some parameters which influenced the size and morphology of the particles have been investigated and optimized, including temperature, pressure, the drug concentration and the solution flow rate. GTW nanoparticles with a minimum MPS of 77.5 nm were successfully prepared from ethanol by a SAS process when the temperature was 63.5 °C, pressure was 29.4 MPa, the drug concentration was 10.2 mg/mL, the solution flow rate was 5.4 mL/min and the CO_2_ flow rate was 8.5 kg/h, respectively. The morphologies of the unprocessed and processed GTW were determined by SEM. In addition, HPLC, XRD, TG, and FTIR analysis indicated that GTW existed as an amorphous form and no degradation occurred after the SAS process. In the present study, based on the results, we also concluded that GTW nanoparticles significantly decreased adjuvant-induced arthritis, which may be due to the protection provided against interleukin and TNF induced cartilage destruction. Further, SAS process was a powerful methodology for improving the physicochemical properties and anti-inflammatory activity of GTW.

## Figures and Tables

**Table 1.** Experimental design matrix to screen for variables that determine the mean particle size (MPS) of GTW and ANOVA results a.

Run	BBD experiments					ANOVA					

	*X*_1_	*X*_2_	*X*_3_	*X*_4_	*Y*	Source	Sum of squares	Degree of freedom	Mean square	*F*-value	*p*-value
1	25	45	20	7	112.9	Model b	5931.62	14	423.69	6.65	0.0005 ***
2	25	55	10	3	123.8	*X*_1_	11.41	1	11.41	0.18	0.6787
3	35	55	20	3	127.8	*X*_2_	578.24	1	578.24	9.07	0.0093 **
4	25	45	30	5	106.8	*X*_3_	486.41	1	486.41	7.63	0.0153 *
5	35	65	20	5	93.6	*X*_4_	536.00	1	536.00	8.41	0.0116 *
6	15	55	10	5	92.6	*X*_1_*X*_2_	642.62	1	642.62	10.08	0.0067 **
7	25	65	30	5	127.9	*X*_1_*X*_3_	11.56	1	11.56	0.18	0.6767
8	25	55	20	5	87.4	*X*_1_*X*_4_	68.89	1	68.89	1.08	0.3161
9	25	65	10	5	83.5	*X*_2_*X*_3_	1204.09	1	1204.09	18.89	0.0007 ***
10	25	45	20	3	121.8	*X*_2_*X*_4_	72.25	1	72.25	1.13	0.3050
11	25	55	20	5	84.1	*X*_3_*X*_4_	506.25	1	506.25	7.94	0.0137 *
12	25	55	20	5	81.5	*X*_1_^2^	792.49	1	792.49	12.44	0.0034 **
13	15	45	20	5	100.7	*X*_2_^2^	880.87	1	880.87	13.82	0.0023 **
14	25	55	30	3	110.4	*X*_3_^2^	602.89	1	602.89	9.46	0.0082 **
15	15	65	20	5	122.4	*X*_4_^2^	541.98	1	541.98	8.50	0.0113 *
16	25	55	20	5	91.3	Residual	892.22	14	63.73		
17	35	45	20	5	122.6	Lack of fit	794.23	10	79.42	3.24	0.1341
18	35	55	20	7	102.2	Pure error	97.99	4	24.50		
19	25	65	20	7	97.0	Cor total	6823.84	28			
						
20	35	55	30	5	114.1	Credibility analysis of the regression equations					
						
21	35	55	10	5	98.1	Index mark				*Y*	
22	15	55	30	5	115.4	Standard deviation				7.98	
23	25	45	10	5	131.8	Mean				104.73	
24	15	55	20	3	112.3	Coefficient of variation %				7.62	
25	15	55	20	7	103.3	Press				4727.88	
26	25	55	30	7	110.5	*R*^2^				0.8692	
27	25	55	10	7	78.9	Adjust *R*^2^				0.7385	
28	25	55	20	5	93.5	Predicted *R*^2^				0.3072	
29	25	65	20	3	88.9	Adequacy precision				9.1724	

a The results were obtained with Design Expert 7.0 software;

b *X*_1_ is precipitation pressure (MPa), *X*_2_ is the precipitation temperature (°C), *X*_3_ is the drug concentration (mg/mL), *X*_4_ is the drug solution flow rate (mL/min) and *Y* is MPS of GTW (nm);

* *p* < 0.05, significant;

** *p* < 0.01, highly significant;

*** *p* < 0.001, extremely significant.

**Table 2.** Contents of GTW and triptolide in unprocessed and processed samples.

Sample	GTW (%) ± SD (*n* = 3)	Triptolide (‰) ± SD (*n* = 3)
Unprocessed	0.52 ± 0.02	0.82 ± 0.03
Processed	0.54 ± 0.02	0.83 ± 0.04

**Table 3.** Effects of processed and unprocessed GTW on inflammatory mediators in Freund’s Complete Adjuvant (FCA)-induced arthritic rats a.

Parameters	Vehicle control	Arthritic control	Unprocessed	Processed
Interleukin-1α (IL-1α) (pg/mL)	2.9 ± 0.44	15.8 ± 0.61	9.3 ± 1.23	6.2 ± 0.62
Interleukin-1β (IL-1β) (pg/mL)	3.7 ± 0.77	27.1 ± 0.66	13.7 ± 0.72	9.9 ± 0.38
Tumour necrosis factor-α (TNF-α) (pg/mL)	4.8 ± 0.76	17.9 ± 0.91	10.2 ± 0.44	6.3 ± 0.54

a Values are presented as mean ± SD, *n* = 6.
